# Supplementary material for: ALMS1-IT1: A Key Player in the Novel Disulfidptosis-Related LncRNA Prognostic Signature for Head and Neck Squamous Cell Carcinoma
Source: Biomolecules. 2024 Feb 23;14(3):266. doi: 10.3390/biom14030266 (PMC10968447; doi:10.3390/biom14030266)
Supplement: Supplementary file 1 [file biomolecules-14-00266-s001.zip › Supplementary Materials.pdf]

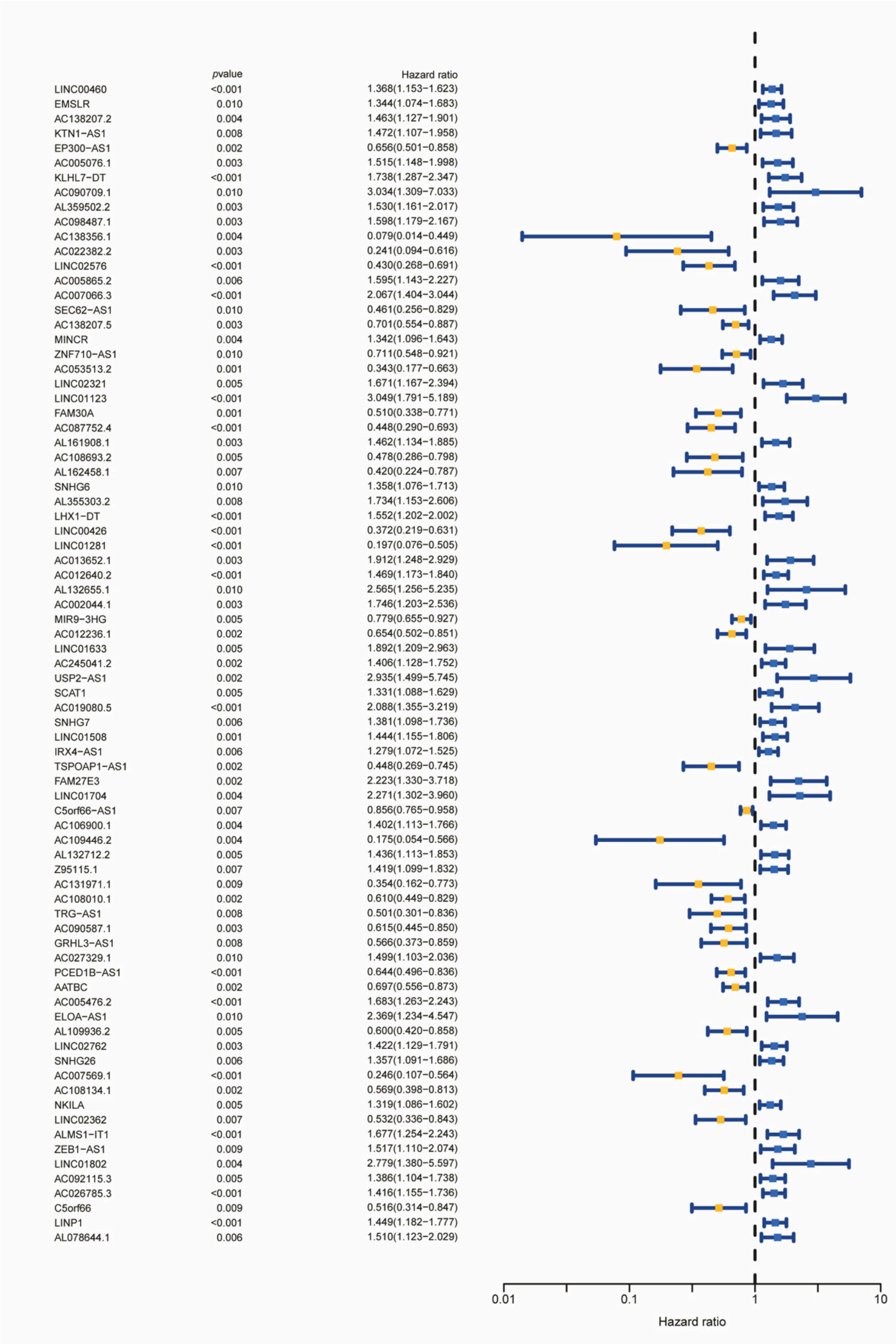

Figure S1. Univariate Cox regression analysis of 1451 DRLs to identify 79 DRLs as prognostic factors.

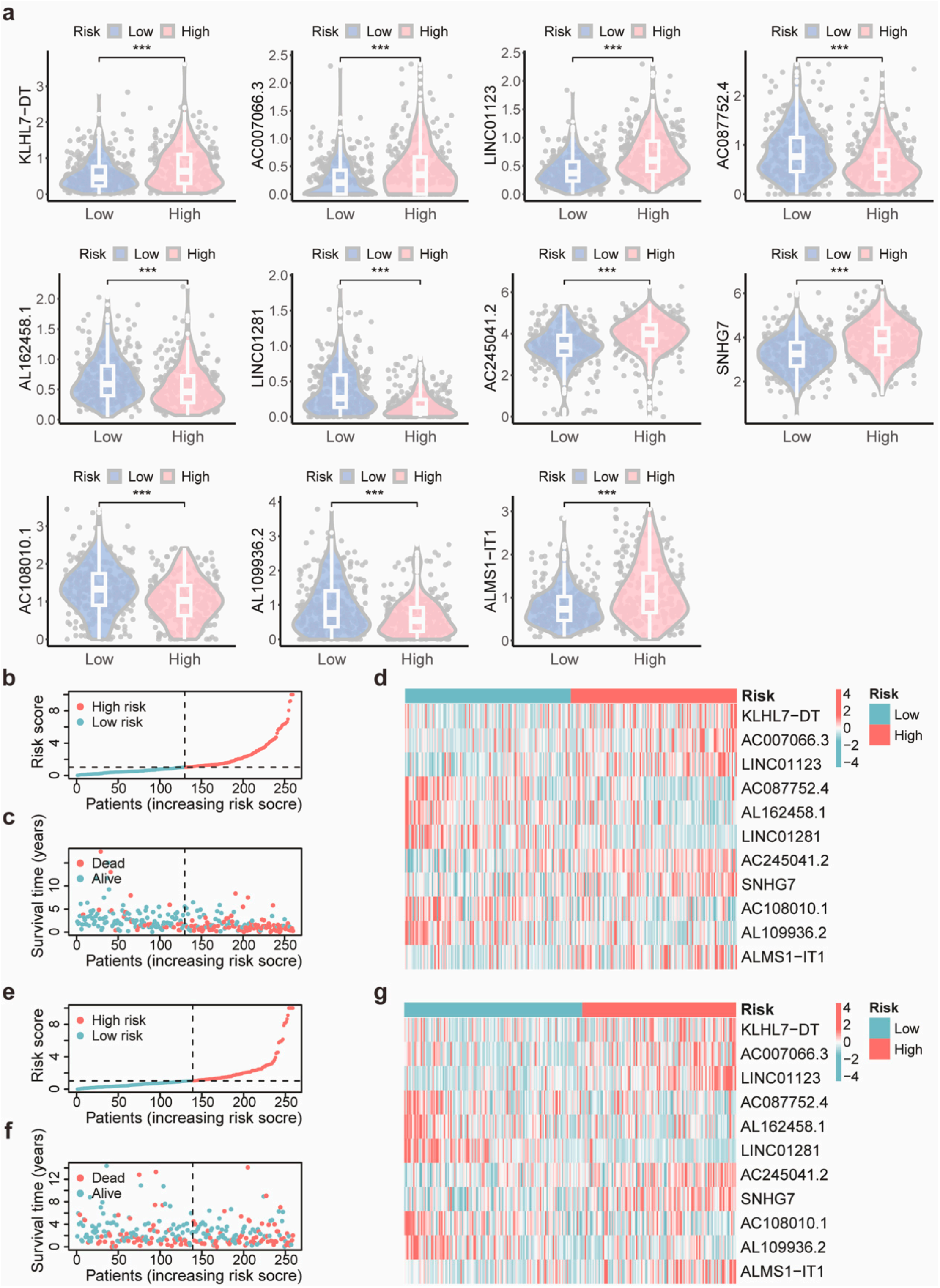

Figure S2. The expression levels of 11 model DRLs. (**a**) The expression levels of 11 model DRLs between low- and high-risk cohorts in overall group. (**b–d**) The risk curve (**b**), survival status (**c**) and 11 model DRLs expression heatmap (**d**) of training cohort. (**e–g**) The risk curve (**e**), survival status (**f**) and 11 model DRLs expression heatmap (**g**) of test cohort. ***, $p < 0.001$.

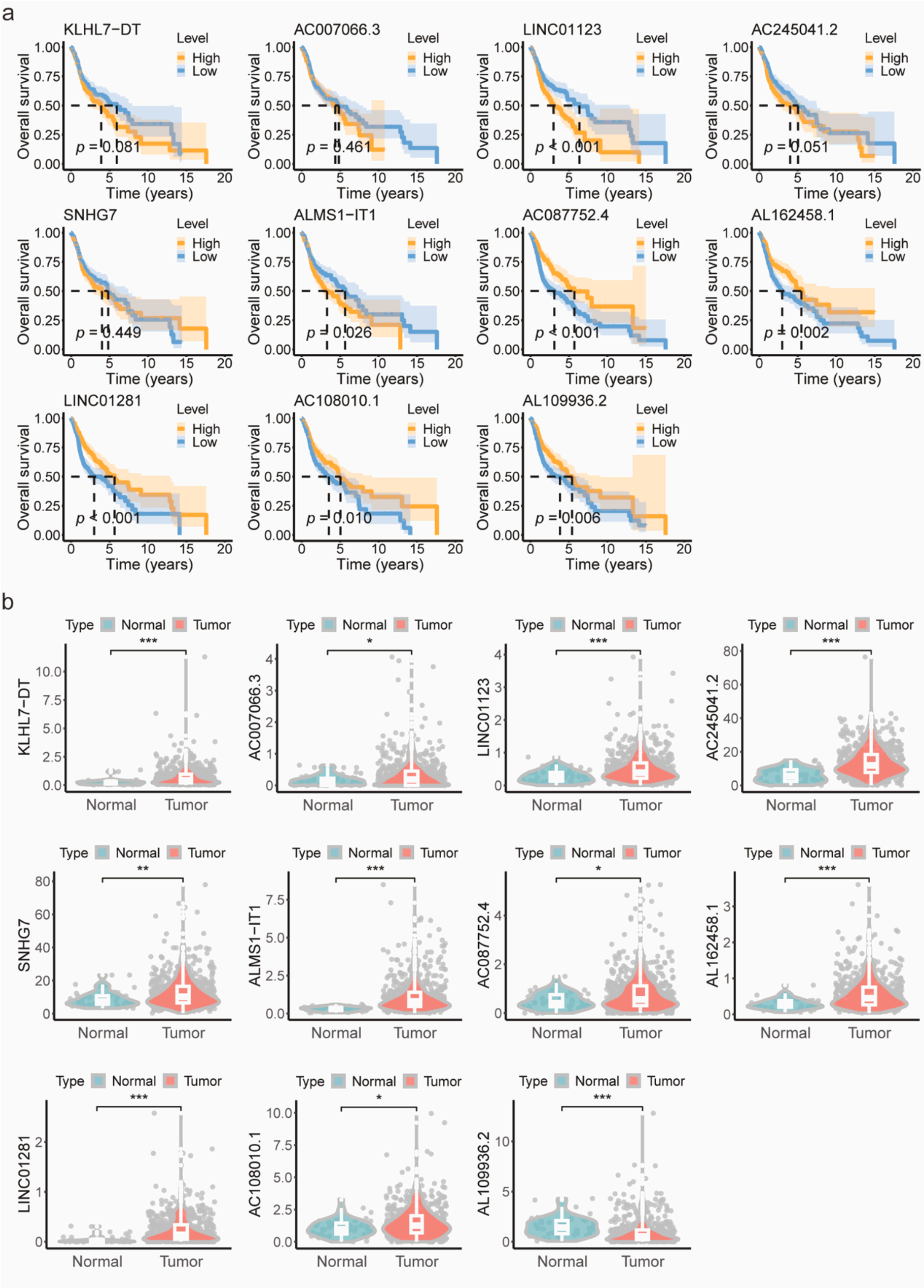

Figure S3. Evaluation of independent prognostic potentials of 11 model DRLs. (**a**) The K-M survival curves between low- and high-level clusters of 11 model DRLs, respectively. (**b**) The expression levels in normal and tumor samples of 11 model DRLs, respectively. *, $p < 0.05$; **, $p < 0.01$; ***, $p < 0.001$.

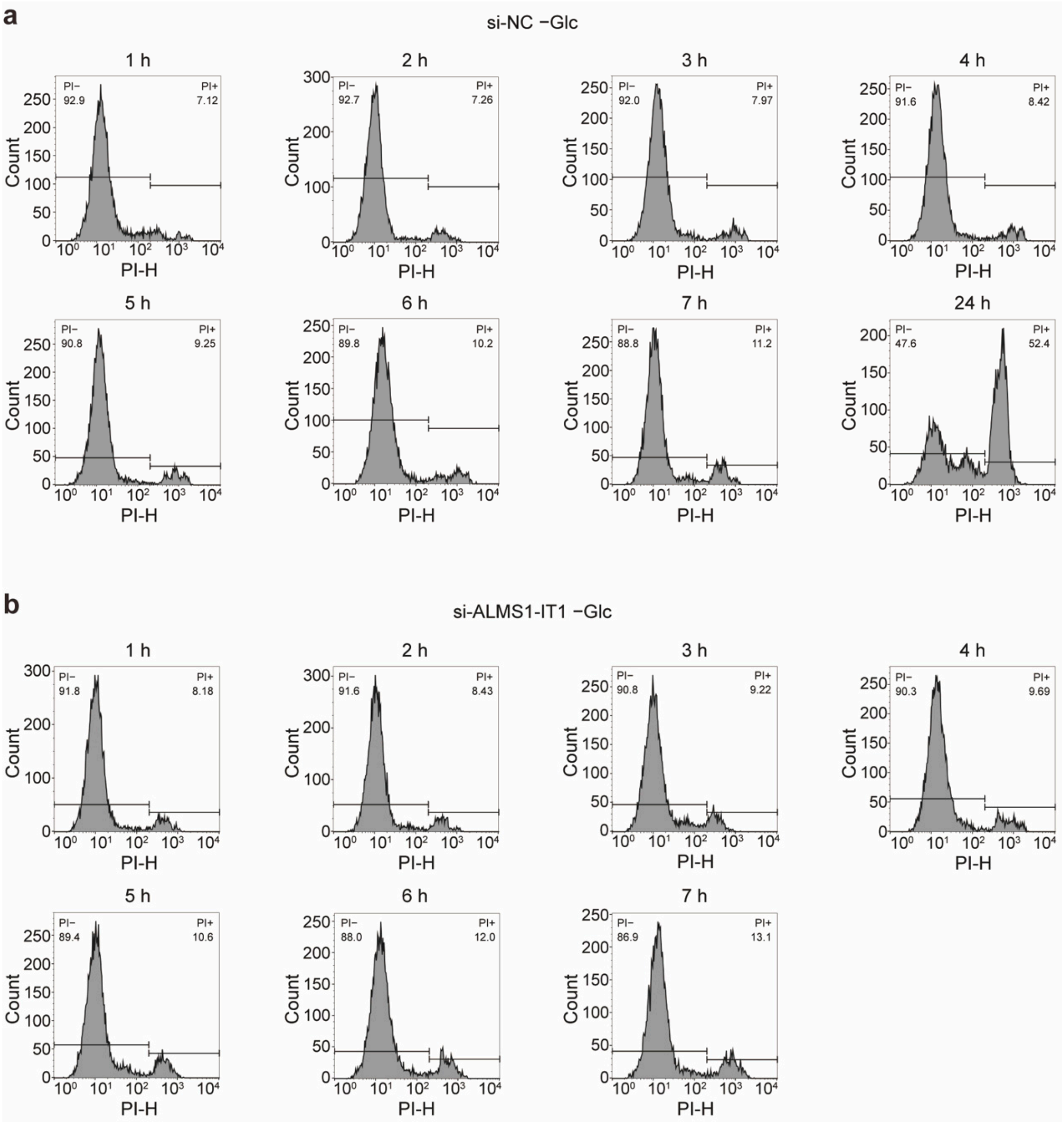

Figure S4. ALMS1-IT1 regulated cell death under glucose starvation. (**a**) Histograms to show the ratios of PI+/PI− cells in negative control (si-NC) HN-6 cells at 1 h, 2 h, 3 h, 4 h, 5 h, 6 h, 7 h and 24 h after cultured in glucose-free medium, respectively. (**b**) Histograms to show the ratios of PI+/PI− cells in si-ALMS1-IT1_#3 HN-6 cells at 1 h, 2 h, 3 h, 4 h, 5 h, 6 h and 7 h after cultured in glucose-free medium, respectively.

Table S1. siRNAs used in this study.

siRNAs	Sequences (5′ to 3′)
si-ALMS1-IT1_#1	GTACTTCACTATACTGTCA
si-ALMS1-IT1_#2	GAGGCACATTATGTTGATT
si-ALMS1-IT1_#3	GGAGAGATTCTTTGAGACT

Table S2. Primers for RT-qPCR in this study.

Primers	Sequences (5′ to 3′)
SLC7A11	Forward: TCCTGCTTTGGCTCCATGAACG
	Reverse: AGAGGAGTGTGCTTGCGGACAT
ALMS1-IT1	Forward: TCAACCATCTTGTGTTTCACCATC
	Reverse: ATGGGTTCCCTAAGGACAAGG
GLUT1	Forward: TTGCAGGCTTCTCCAACTGGAC
	Reverse: CAGAACCAGGAGCACAGTGAAG
HK1	Forward: CTGCTGGTGAAAATCCGTAGTGG
	Reverse: GTCCAAGAAGTCAGAGATGCAGG
HK2	Forward: GAGTTTGACCTGGATGTGGTTGC
	Reverse: CCTCCATGTAGCAGGCATTGCT
HKDC1	Forward: ATCGCCGACTTCCTGGACTACA
	Reverse: GCCTTGAAACCTTTGGTCCACC
H6PD	Forward: GGTGGACCATTACTTAGGCAAGC
	Reverse: CTTCAGCATCCACGGTCTCTTTC
G6PD	Forward: CTGTTCCGTGAGGACCAGATCT
	Reverse: TGAAGGTGAGGATAACGCAGGC
PGLS	Forward: TGTGGCAACTGGAGAAGGCAAG
	Reverse: CTCGTCCAAGAACCAGCACAGT
PGD	Forward: GTTCCAAGACACCGATGGCAAAC
	Reverse: CACCGAGCAAAGACAGCTTCTC
RPIA	Forward: GTATGGCTTGACCCTCAGTGATC
	Reverse: CAGCCACAATCTTCTCCTGGGT
RPE	Forward: AAGCCAATGGCTGTAGCAGGAG
	Reverse: CTGAGGTTCCTGGTTTGATGGC
TKT	Forward: CCAAGTGATGGCGTTGCTACAG
	Reverse: TTGTCCGACCTGGAAGTCCTCA
TALDO1	Forward: TGCCTGTGCTCTCAGCCAAGG
	Reverse: TTCTCCACAGCCATCTGGTCCT
PRPS1	Forward: GGCTGACACTTGTGGCACAATC
	Reverse: GATGCGAGAAATAGCAGGACCG
PRPS1L1	Forward: CCAGCCATTTCTCGCATCAACAC
	Reverse: GGCTTCTGCAAGGATCATGGAG

PRPS2	Forward: GGTCACGAAGAAGTTCAGCAACC<br>Reverse: GAGGAGTTCCATCAGGTTGTCG
RBKS	Forward: GACAGAACCTGAGCCAAAGCAC<br>Reverse: GGATAGTAAGCCAGGTAGAAGGC
RPL13A	Forward: CTCAAGGTGTTTGACGGCATCC<br>Reverse: TACTTCCAGCCAACCTCGTGAG
ACTB	Forward: CACCATTGGCAATGAGCGGTTC<br>Reverse: AGGTCTTTGCGGATGTCCACGT

Table S3. Antibodies used for WB in this study.

Antibodies	Source	Catalog Number	Dilution
MYH9	Proteintech, Wuhan, China	11128-1-AP	1:5000
TLN1	Proteintech, Wuhan, China	14168-1-AP	1:5000
Drebrin	Proteintech, Wuhan, China	10260-1-AP	1:5000
Pan-Actin	Beyotime, Shanghai, China	AG8001	1:1000
HRP-conjugated Affinipure Goat Anti-Rabbit IgG(H+L)	Proteintech, Wuhan, China	SA00001-2	1:10,000

Table S4. Reagents used in this study

Reagents	Source	Catalog Number
*Trans*IT-X2® Dynamic Delivery System	Mirus Bio, WI, USA	MIR 6000
SteadyPure Quick RNA Extraction Kit	Accurate Biotechnology (Hunan) Co., Ltd., Changsha, China	AG21023
Evo M-MLV RT Mix Tracking Kit with gDNA Clean for qPCR (Yellow)	Accurate Biotechnology (Hunan) Co., Ltd., Changsha, China	AG11734
SYBR Green Premix *Pro Taq* HS qPCR Tracking Kit (Rox Plus)	Accurate Biotechnology (Hunan) Co., Ltd., Changsha, China	AG11735
Propidium Iodide Staining Solution (PI)	BD Biosciences, NJ, USA	51-66211E, 556547
10× Binding Buffer	BD Biosciences, NJ, USA	51-66121E, 556547
NuPAGE® LDS sample buffer (4×)	Life Technologies, MA, USA	NP0007
BeyoGel™ sample reducing agent (10×)	Beyotime, Shanghai, China	P0733
$NADP^+$/NADPH Assay Kit with WST-8	Beyotime, Shanghai, China	S0179

2-Deoxy-D-glucose (2-DG)	MedChemExpress (MCE), NJ, USA	HY-13966
TCEP hydrochloride	MedChemExpress (MCE), NJ, USA	HY-W011500
BAY-876	MedChemExpress (MCE), NJ, USA	HY-100017
Glutor	Sigma-Aldrich, MO, USA	SML2765
4% paraformaldehyde	Beyotime, Shanghai, China	P0099
Actin-Tracker Red-594	Beyotime, Shanghai, China	C2205S
Immunofluorescence Staining Secondary Antibody Dilution Buffer	Beyotime, Shanghai, China	P0108
Antifade Mounting Medium with DAPI	Beyotime, Shanghai, China	P0131

Table S5. The clinical characteristics of TCGA-HNSC patients in the training and test group.

Covariates	Type	Total	Train	Test	Pvalue
**Age**	<=65	341(65.7%)	177(68.08%)	164(63.32%)	0.2942
	>65	178(34.3%)	83(31.92%)	95(36.68%)	
**Gender**	Male	383(73.8%)	194(74.62%)	189(72.97%)	0.7447
	Female	136(26.2%)	66(25.38%)	70(27.03%)	
**Stage**	Stage I	20(3.85%)	6(2.31%)	14(5.41%)	0.1104
	Stage II	97(18.69%)	56(21.54%)	41(15.83%)	
	Stage III	105(20.23%)	49(18.85%)	56(21.62%)	
	Stage IV	283(54.53%)	144(55.38%)	139(53.67%)	
	unknow	14(2.7%)	5(1.92%)	9(3.47%)	
**T**	T1	35(6.74%)	14(5.38%)	21(8.11%)	0.611
	T2	150(28.9%)	78(30%)	72(27.8%)	
	T3	135(26.01%)	68(26.15%)	67(25.87%)	
	T4	183(35.26%)	95(36.54%)	88(33.98%)	
	unknow	16(3.08%)	5(1.92%)	11(4.25%)	
**N**	N0	243(46.82%)	129(49.62%)	114(44.02%)	0.2886
	N1	83(15.99%)	36(13.85%)	47(18.15%)	
	N2	162(31.21%)	86(33.08%)	76(29.34%)	
	N3	9(1.73%)	3(1.15%)	6(2.32%)	
	unknow	22(4.24%)	6(2.31%)	16(6.18%)	
**M**	M0	488(94.03%)	251(96.54%)	237(91.51%)	0.1997
	M1	6(1.16%)	1(0.38%)	5(1.93%)	
	unknow	25(4.82%)	8(3.08%)	17(6.56%)	
