# Supplementary figures and images for: ALMS1-IT1: A Key Player in the Novel Disulfidptosis-Related LncRNA Prognostic Signature for Head and Neck Squamous Cell Carcinoma
Source: Biomolecules. 2024 Feb 23;14(3):266. doi: 10.3390/biom14030266 (PMC10968447; doi:10.3390/biom14030266)

Figure 8a\_2

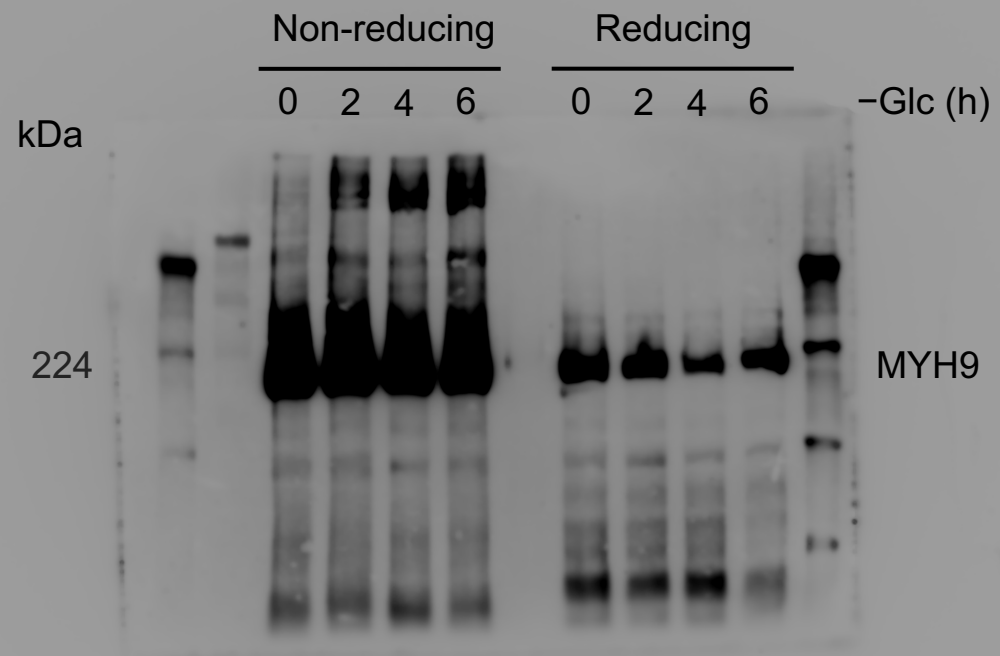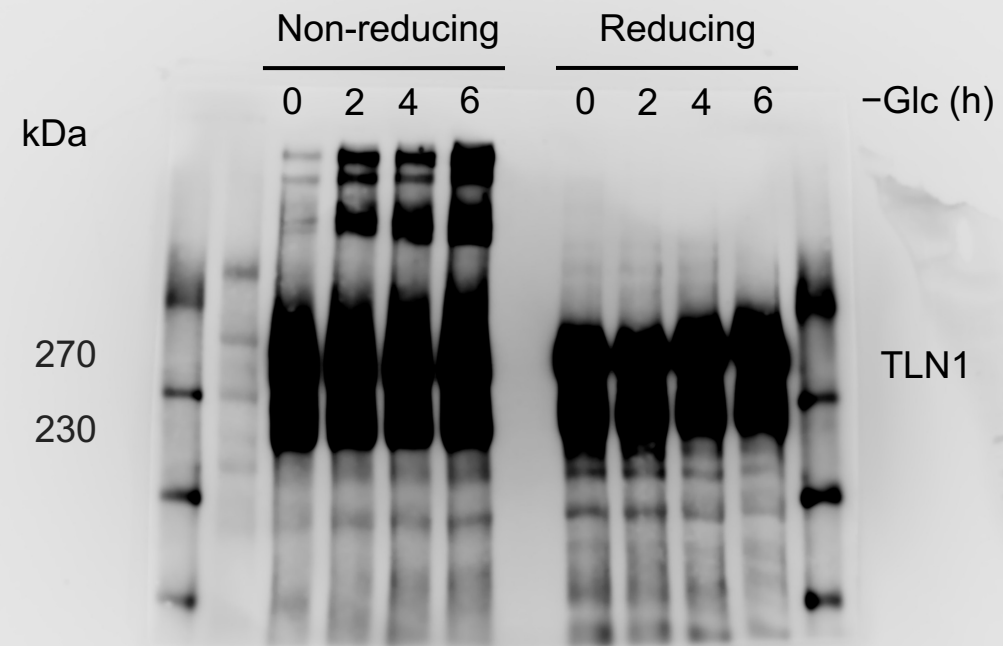

Figure 8a\_3

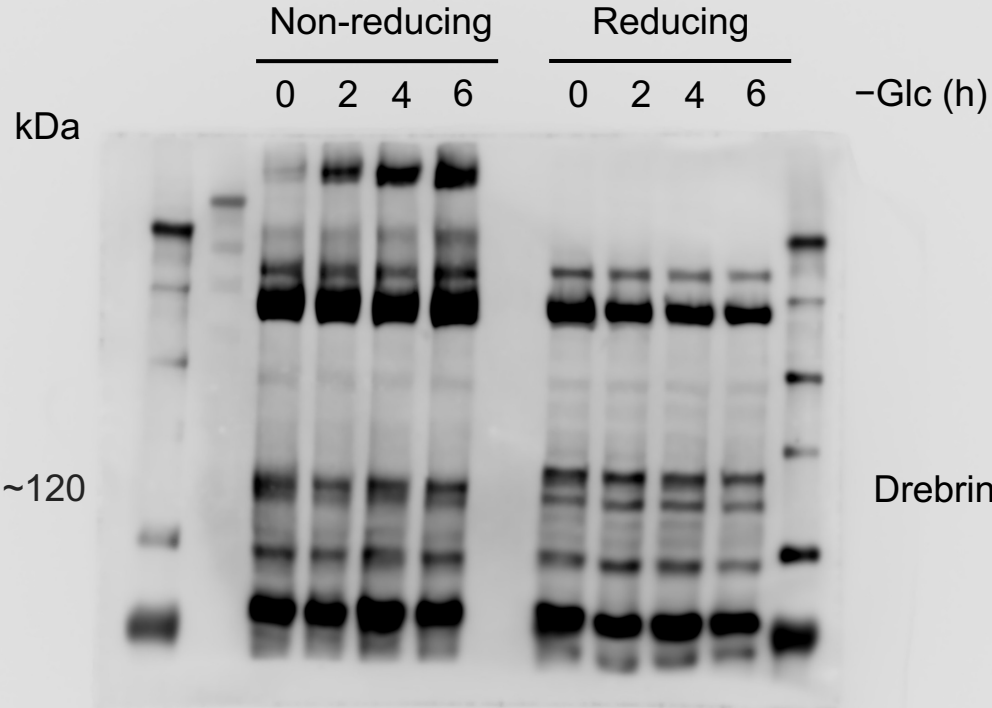

Figure 8a\_4

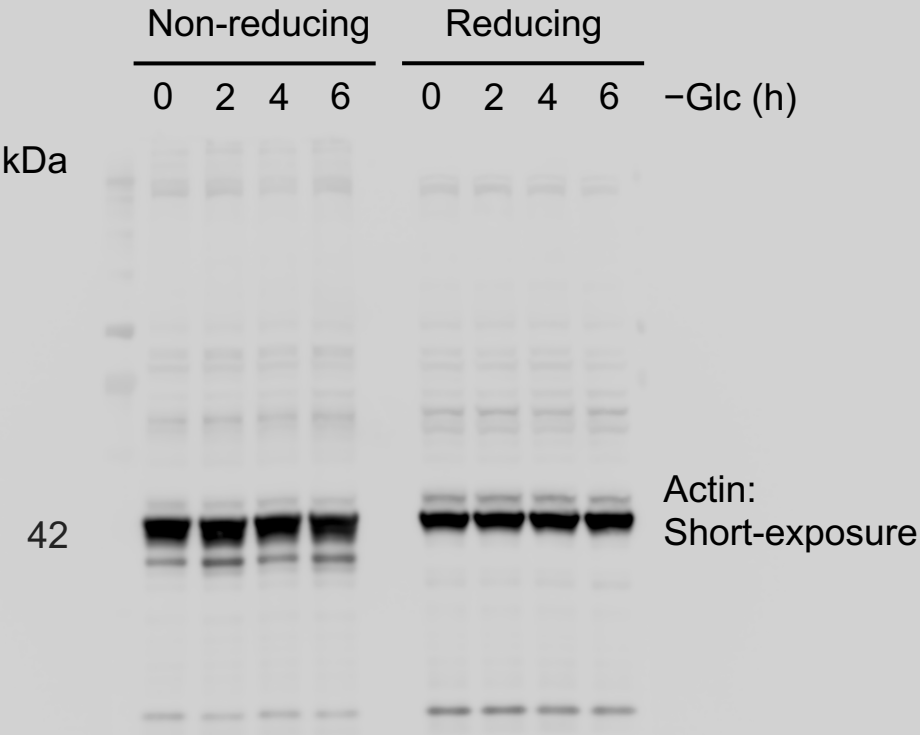

Figure 8a\_5

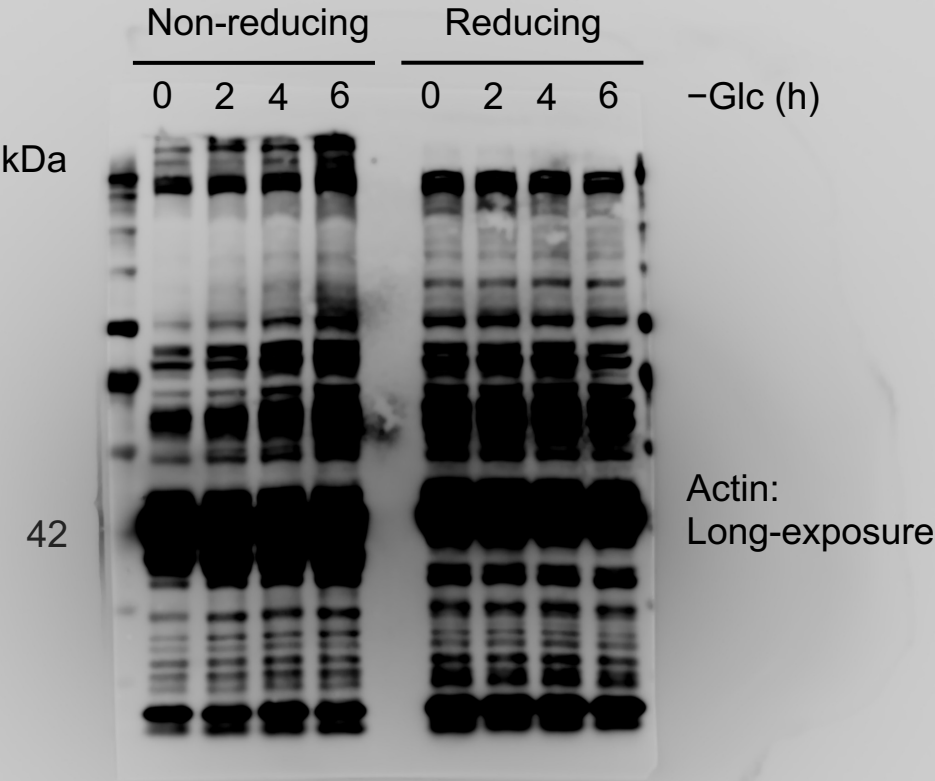

Figure 8a\_6

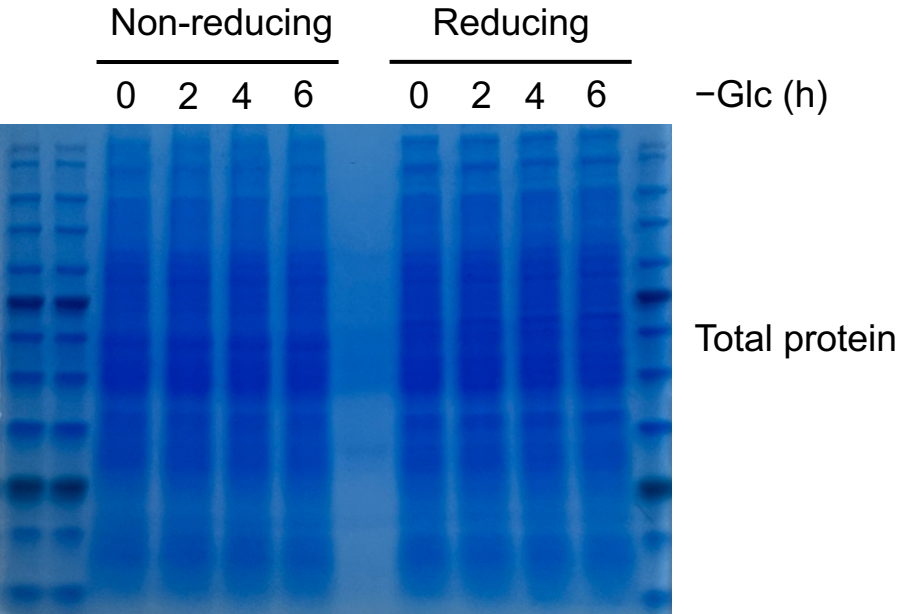

Figure 8b\_1

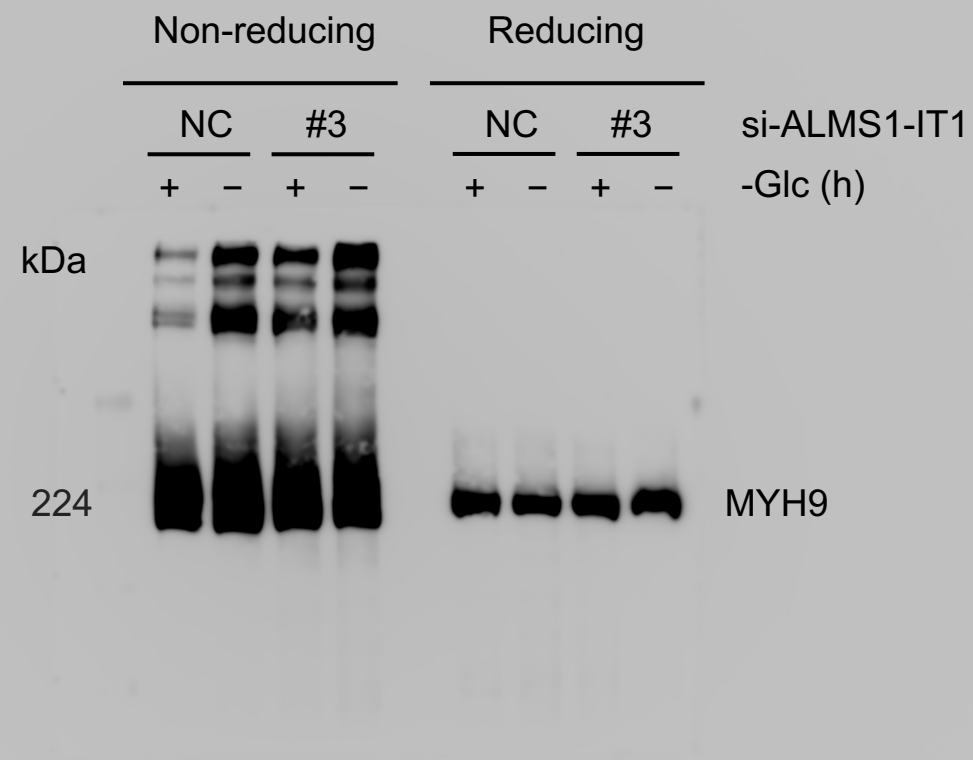

Figure 8b\_2

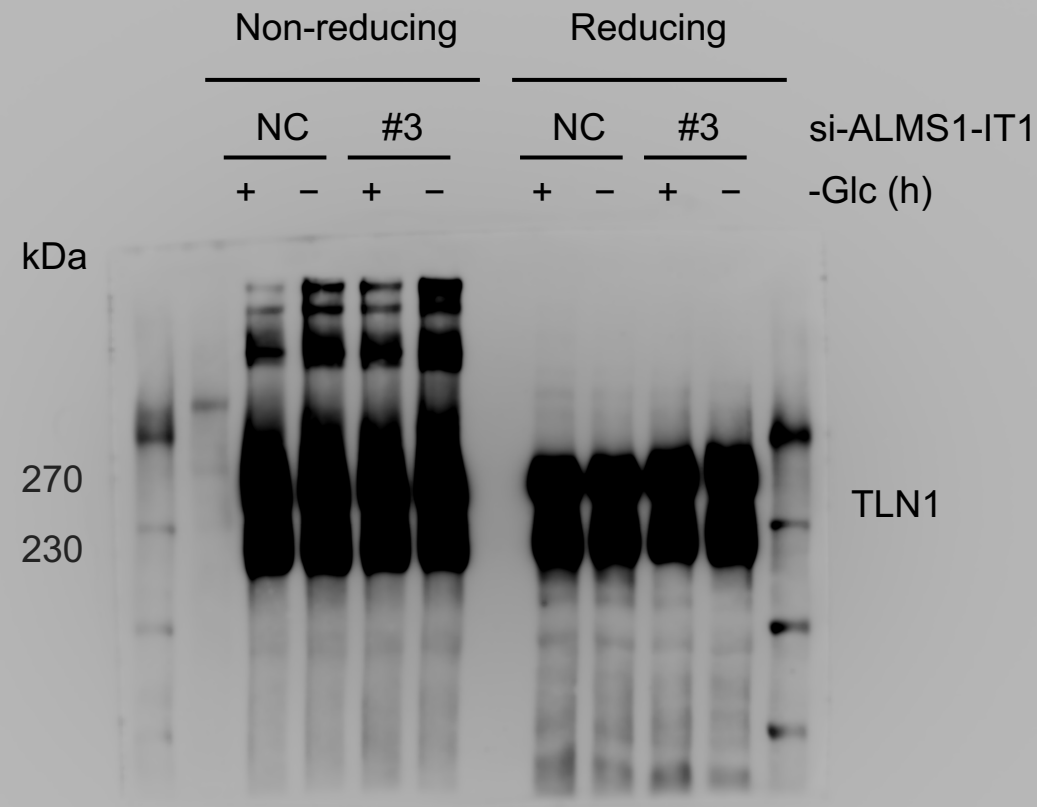

Figure 8b\_3

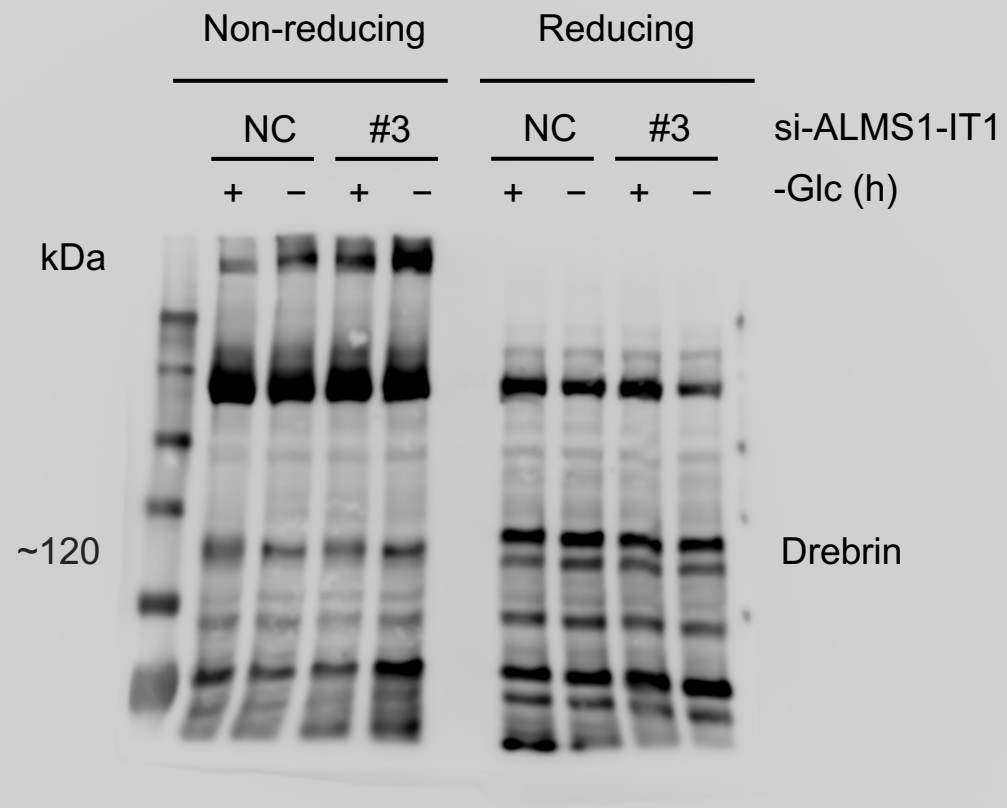

Figure 8b\_4

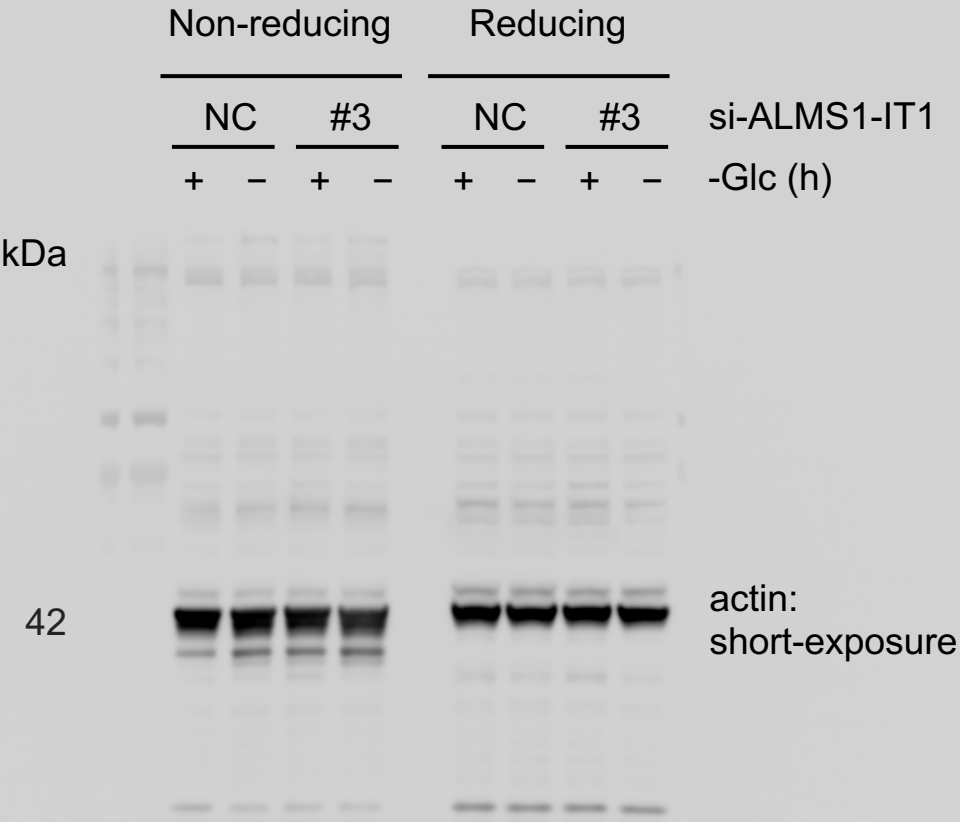

Figure 8b\_5

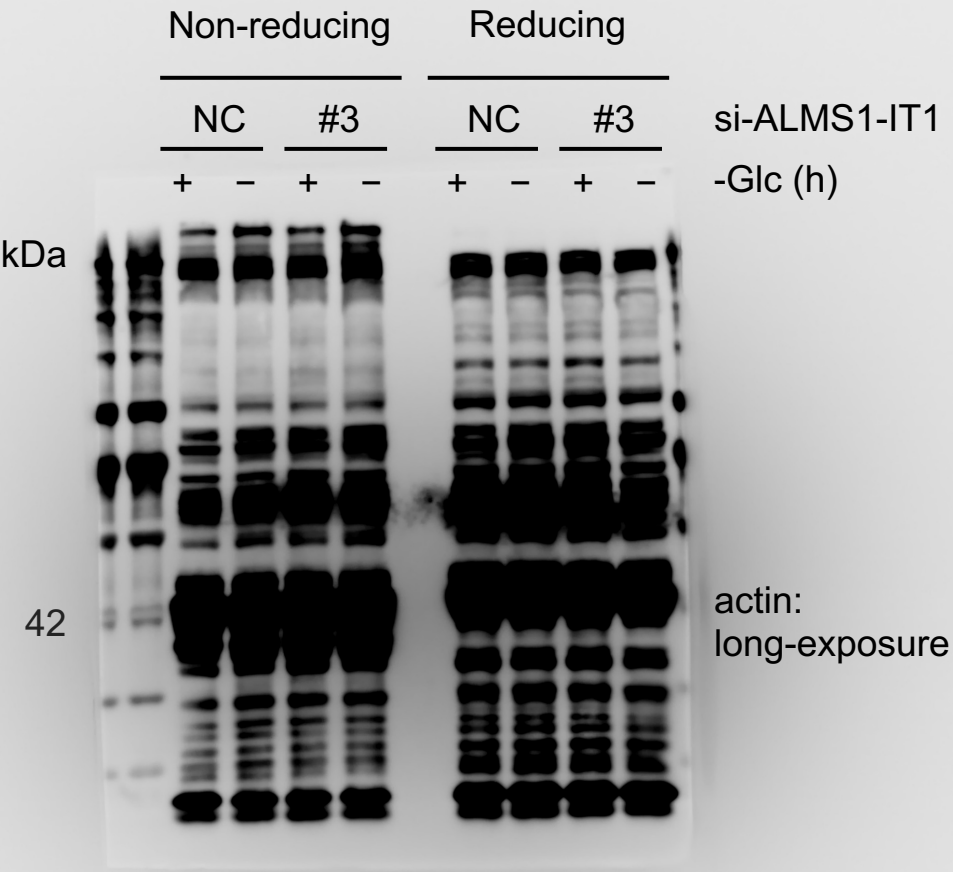

Figure 8b\_6

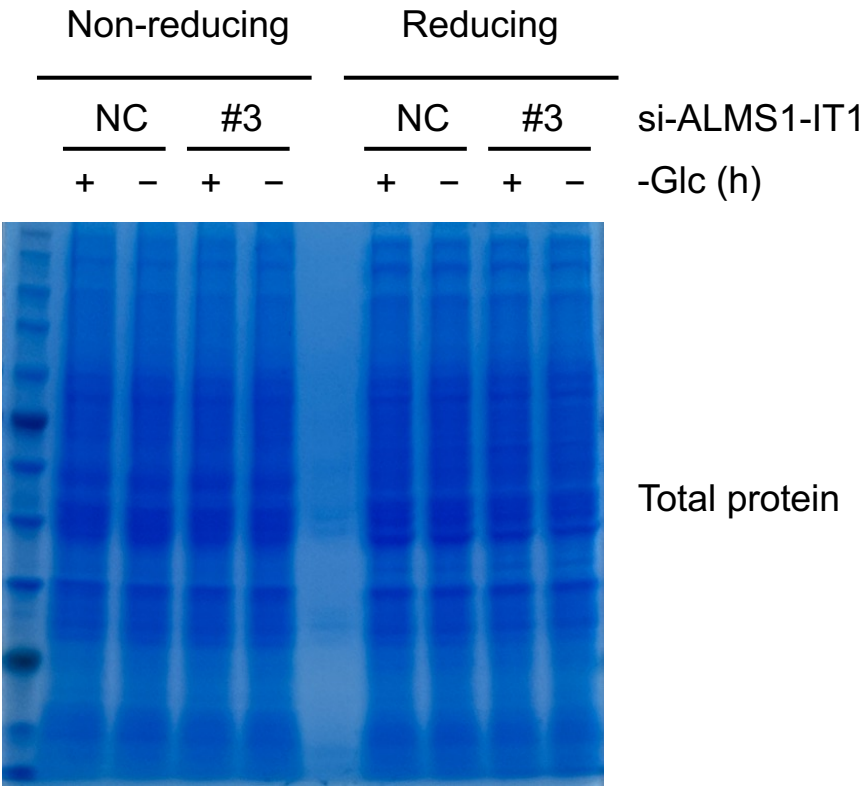

Supplement: Supplementary file 1 [file biomolecules-14-00266-s001.zip › original images.pdf]
